# Antibiotic prescribing trends in a pediatric population in Lithuania in 2003–2012: Observational study

**DOI:** 10.1097/MD.0000000000017220

**Published:** 2019-11-15

**Authors:** Egle Karinauske, Skaiste Kasciuskeviciute, Vilma Morkuniene, Kristina Garuoliene, Edmundas Kadusevicius

**Affiliations:** a Department of Clinical Pharmacology, Institute of Physiology and Pharmacology, Lithuanian University of Health Sciences; b Kaunas University of Applied Science, Kaunas; c Ministry of Health of the Republic of Lithuania, Vilnius, Lithuania.

**Keywords:** antibiotics, macrolides, pediatric, prescription rate, utilization

## Abstract

The purpose of this study was to determine the trends in consumption of antibiotics and evaluate the antibiotic prescription rates in the pediatric population in Lithuania during 2003 to 2012.

A cross-sectional study. Data of systemic antibiotic use in pediatric population for outpatient treatment was derived from National Health Insurance Fund database. Consumption was expressed as WHO ATC defined daily dose (DDD)/1000 children/day and as a number of prescriptions written in the general population per year. Statistical analysis was performed using the SPSS/W 20.0 software (Statistical Product and Service Solutions for Windows).

Total utilization of antibiotics (expressed in DDD units) during study period increased by 8.40% (from 5.67 to 6.19 DDD/1000 children/day) and by 5.96% expressed in prescription rate (from 585.84 to 622.97 prescriptions/1000 children/year). The most popular antibiotic group was macrolides which showed the highest increase of utilization 5.9 times (from 0.27 DDD/1000 children/day in 2003 to 1.66 DDD/1000 children/day in 2012).

The most common indications for antibiotic prescribing for children in 2012 were acute bronchitis (25.6%), acute tonsillitis (21.7%) and acute pharyngitis (14.6%). Amoxicillin had the highest probability to be chosen to treat acute tonsillitis (prob. [probability] = .2875) and acute pharyngitis (prob. = .5553). Clarithromycin had the highest probability to be chosen to treat acute bronchitis (prob. = .4222).

Most of the diseases treated with antibiotics were viral infections. The most commonly prescribed antibiotics were broad-spectrum. The consumption of antibiotics was evenly increasing during 2003 to 2012 period, but the distribution of separate antibiotic group remained the same.

## 1 Introduction

Antibiotics are the most commonly prescribed medicines in children's population across the Europe,^[1]^ including Lithuania. The most frequent illnesses which are treated with antibiotics are acute respiratory infections^[2]^ and acute otitis media. It is thought that a minority of infections are caused by bacteria. The most of upper respiratory, ear and nose infections are caused by viruses and are unlikely to benefit from antibiotic treatment.^[3]^

The overuse and misuse of antibiotics shows increasing resistance to pathogens. The resistance of antibiotics has become a global public health problem in 21st century. The European Surveillance of Antibiotic Consumptions (ESAC) and the European Antimicrobial Resistance Surveillance Network (EARS) collect data on antibiotic consumption and resistance in European countries.^[4]^ It is thought the reason for prescribing different antibiotics according to national guidelines is that pathogens are widely distributed in European countries. For example, the low prescribing rates and the selection of narrow spectrum antibiotics are associated with northern European countries and high prescribing rates and use of broad-spectrum antimicrobial drugs are shown in southern European countries.^[4,5]^ Some countries noticed this irrational use of antibiotics and established systems to fight the overuse and misuse of antibiotics, for example, Sweden's nationwide government-funded multidisciplinary program (STRAMA)^[1]^ and a similar national campaign was developed in France in 2001.^[6]^ During their campaign they have established national guidelines for the management of respiratory tract infections which were published in 2005.^[6]^

The clinical research was performed among Kaunas district General Practitioners (GP's).^[7]^ Their aim was to identify the rate and causality of prescriptions of antibiotics for upper respiratory tract infections (URTI). This study has shown that GP's prescribed antibiotics for 39.3% of patients suffering from URTI, 25.6% of these patients had common cold. Other 36.7% of patients expected to get a prescription for antibiotics but there was no indication. The 24% of patients started using antibiotics by themselves without the prescription.^[7]^ This study helps to understand the need of national campaigns with should promote rational use of antibiotics, especially when treating children.

We acknowledged that there is a need of pharmacoepidemiological researches in the pediatric population in Lithuania. The purpose of this study was to determine the trends in consumption of antibiotics and evaluate the antibiotic prescription rates in the pediatric population in Lithuania during 2003 to 2012. This study can provide baseline information on the recent status of antibiotics overuse or misuse in the pediatric population in Lithuania for developing public health policies to manage antimicrobial usage.

## 2 Materials ant methods

### 2.1 Data source

The data was obtained from the National Health Insurance Fund (NHIF) database over the 10-year period (2003–2012). The NHIF is responsible for auditing the consumption of reimbursed medicines. The database contains information about patient, disease, prescription of antibiotic in the Anatomical Therapeutic Chemical (ATC) code, pack size and strength, name of the active substance, name and cost of medication.

### 2.2 Analysis

The cross-sectional study was conducted in January 2003 to December 2012. For this study data of systemic antibiotic use in pediatric population for outpatient treatment was obtained from NHIF database. We identified all antibacterial drugs for systemic use (ATC code J01, available online: https://www.whocc.no/atc_ddd_index/?code=J01) prescribed for children (0–18 years). Systemic antibacterial drugs were divided into 6 antibiotic subgroups according to ATC codes: beta-lactamase sensitive penicillins (J01CE), penicillins with extended spectrum (J01CA), combinations of penicillins, including beta-lactamase inhibitors (J01CR), cephalosporins (J01DB, J01DC, J01DD, J01DE) and macrolides (J01FA); the remaining antibiotics which did not fit in the mentioned subgroups were pooled in the subgroup called *others* (J01A, J01G, J01MA, J01E).

The consumption was expressed as World Health Organization (WHO) ATC defined daily dose (DDD) /1000 children/day and as a number of prescriptions written in the general population per year.

Main outcomes and measures: all the utilization data were converted to DDDs per 1000 children per day (DDD/1000 children/day). Due to a large variation of body weight in pediatric population^[8]^ and in order to exclude WHO DDD methodology limitation to pediatrics population, we used a number of prescriptions for systemic antibiotics (ATC code J01) per 1000 children.

Statistical analysis was performed using the SPSS/W 20.0 software (Statistical Product and Service Solutions for Windows). The descriptive statistics was used to estimate results. The data is presented in absolute numbers of prescribed antibiotics by disease in 2003 to 2012.

The methodical recommendation of URTI diagnostics and treatment and National Institute of Health and Care Excellence (NICE) recommendations were used to evaluate the compliance.^[9,10]^

### 2.3 Ethical considerations

Data were collected as part of routine surveillance; therefore, the study was exempt from review by an ethics board.

## 3 Results

### 3.1 Antibiotic utilization by DDD/1000 children/day method

**Figure 1:**
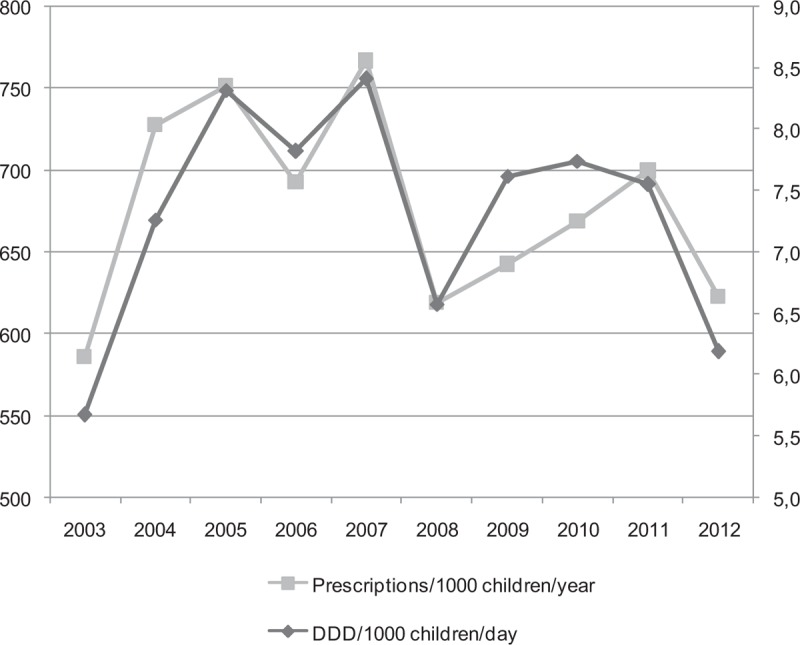
Trends of antibiotic utilization (DDD/1000 children/day and prescriptions/1000 children/year) in the pediatric population in Lithuania during 2003 to 2012.

**Figure 2:**
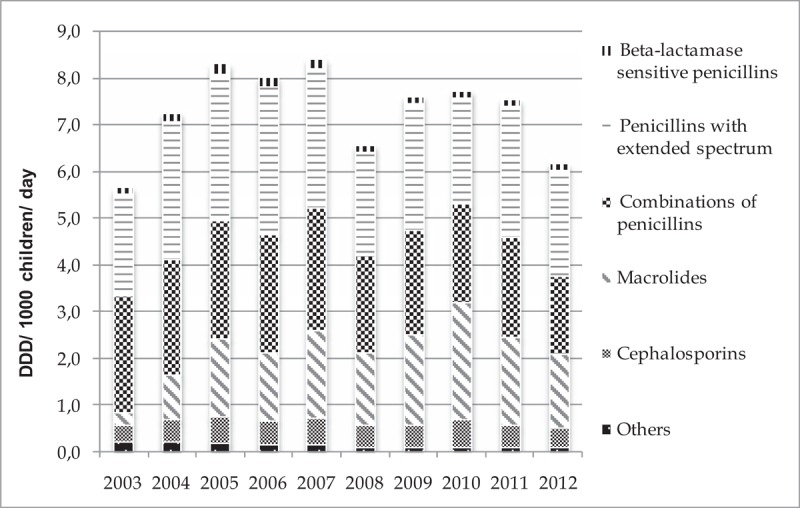
Distribution of antibiotic utilization by subgroups in 2003 to 2012 in Lithuania.

The total utilization of systemic antibiotics (ATC code J01) in pediatric population increased by 8.40% (from 5.67 DDD/1000 children/day in 2003 to 6.19 DDD/1000 children/day in 2012). The maximum value was observed in 2007 (8.41 DDD/1000 children/day) (Fig. 1). The utilization of macrolides showed the highest increase by 5.9 times (from 0.27 DDD/1000 children/day in 2003 to 1.66 DDD/1000 children/day in 2012), while the combination of penicillins, including beta-lactamase inhibitors, decreased by ∼1.5 times. In the remaining (penicillins with extended spectrum, beta-lactamase sensitive penicillins, cephalosporines and other) subgroups the utilization of antibiotics persisted relatively stable in the same study period. The distribution of total utilization in subgroups of antibiotics dispensed by the study years is presented in Figure 2.

All in all, the most frequently used antibiotics were penicillins with extended spectrum (2.67 DDD/1000 children/day), combinations of penicillins, including beta-lactamase inhibitors (2.27 DDD/1000 children/day) and macrolides (1.58 DDD/1000 children/d) in 2007. However, the utilization of beta-lactamase-sensitive penicillins was the smallest (0.17 DDD/1000 children/day) in 2007, comparing to the other defined years (Fig. 2).

### 3.2 Antibiotic utilization by number of prescriptions per 1000 children/year method

**Figure 3:**
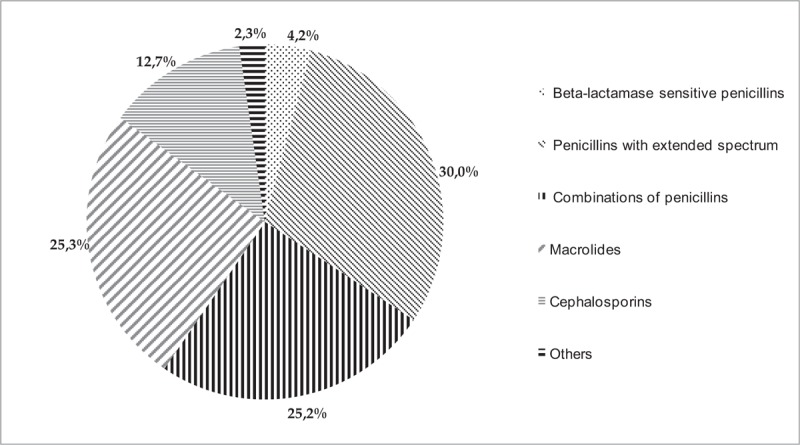
The prescription rate of antibiotics by subgroups in 2011 in Lithuania.

Total antibiotic utilization expressed in prescriptions rate in Lithuania during study period increased by 5.96% from 585.84 to 622.97 prescriptions/1000 children/year (Fig. 1) (pediatric population was 670,040 children in Lithuania in 2011), (Fig. 1). The total antibiotic utilization in 2011 was 698 prescriptions per 1000 children/year (95% CI 697–699). Penicillins with extended spectrum (J01CA) most frequently were prescribed in 2011, ∼211 prescriptions /1000 children/year (30.2% of all the prescriptions in 2011). Prescriptions of the combinations of penicillins, including beta-lactamase inhibitors (J01CR) and macrolides (J01FA) distributed equally 176 prescriptions /1000 children/year (25.25%) in 2011. These 3 systemic antibiotic subgroups were prescribed most frequently and accounted for about 80.7% of all antibiotic prescriptions. Beta-lactamase-sensitive penicillins (J01CE) accounted for only 4.2% (29 prescriptions 1000 children/year) of all prescription. Cephalosporines (J01DB, J01DC, J01DD, J01DE) –89 prescriptions/1000 children/year (12.7%), and all the remaining used antibiotics (subgroup OTHER) were rarely prescribed, for example, accounted only 16 prescriptions per 1000 children/year (2.3%) (Fig. 3).

### 3.3 Prescription of antibiotics due to disease code

The most common indications for antibiotic prescribing for children in 2012 were acute bronchitis (WHO International Classification of Diseases (ICD-10) code J20; 25.6% of all antibiotics prescriptions), acute tonsillitis (J03; 21.7%) and acute pharyngitis (J02; 14.6%). The other diseases for antibiotics prescription were as follows: acute laryngitis and tracheitis (J04; 9.4%), acute rhinopharyngitis (J00; 5.6%), acute sinusitis (J01; 4.7%) and pneumonia (J13-J18; 4.7%), acute laryngopharyngitis (J06; 3.51%), suppurative otitis media (H66; 0.89%).

**Figure 5:**
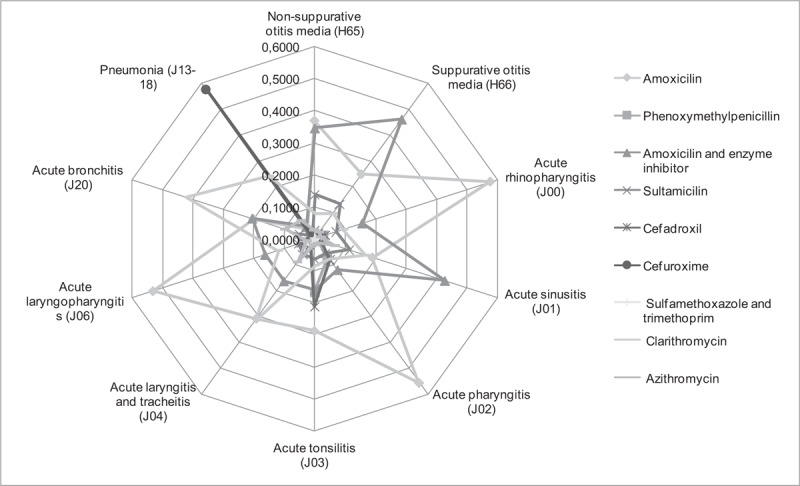
Probability of antibiotics prescription according to disease in Lithuania over 2012 year.

The greatest probability (*P* > .500) to get a prescription of amoxicillin (ATC code J01CR04) was if you are suffering from acute rhinopharyngitis (J00), acute pharyngitis (J02) and acute laryngopharingitis (J06). The greatest probability (prob. > .500) to get a prescription of cefuroxime (J01DC02) is for pneumonia (J13–18) (Fig. 5).

Fifteen different antibiotics were prescribed for patients who suffered from acute bronchitis in Lithuania in 2012. Clarithromycin (J01FA09) was prescribed the most (n = 43,298; 42.02% cases, the probability to choose clarithromycin is prob. = .4222), followed by amoxicillin with clavulanic acid (J01CR02; n = 21,035; 20.42% cases, prob. = .2051) and amoxicillin (n = 20,499; 19.90% cases, prob. = .1999) (see data at Table 1).

**Table 1:**
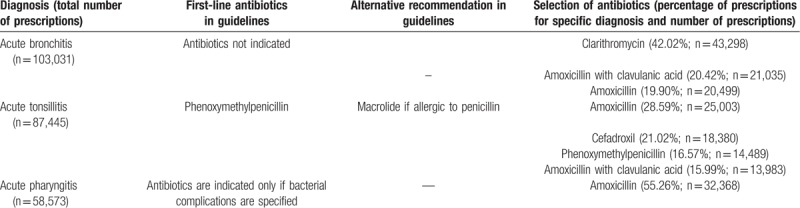
The most commonly prescribed antibiotics 2003 to 2012 years.

Amoxicillin was the most commonly prescribed antibiotic for acute tonsillitis treatment (n = 25,003; 28.59%, prob. = .2875), followed by cefadroxil (J01DB05; n = 18,380; 21.02%, prob. = .2114), phenoxymethylpenicillin (J01CE02; n = 14,489; 16.57%, prob. = .1666) and amoxicillin with clavulanic acid (n = 13,983; 15.99%. prob. = .1608) (see data at Table 1).

Amoxicillin was most commonly prescribed for patients who suffered from acute pharyngitis in Lithuania (n = 32,368; 55.26%, prob. = .5553) (Table 1 and Fig. 5). Also, amoxicillin has the highest probability to be prescribed for treatment of acute rhinopharyngitis (prob. = .5762) and acute laryngopharingitis (prob. = .592). Amoxicillin with clavulanic acid has the highest probability to be prescribed for treatment of suppurative acute otitis media (prob. = .4607) and acute sinusitis (prob. = .4257). Amoxicillin (prob. = .3682) and amoxicillin with clavulanic acid (prob. = .3452) have the greatest probability to be prescribed for treatment of non-suppurative acute otitis media. Clarithromycin (prob. = .3144) and amoxicillin (prob. = .3078) have the highest probability to be prescribed for treatment of acute laryngitis and tracheitis. Cefuroxime has the highest probability to be prescribed for treatment of pneumonia (prob. = .5748) (Fig. 5).

## 4 Discussion

During the study period, the utilization of systemic antibiotics in children remained relatively stable in Lithuania. The utilization of antibiotics in pediatric population in Lithuania was low compared with other European countries. For instance, the total utilization of antibiotics in Lithuania was 2.67 times lower than in Spain^[13]^ in the same period of time.

**Figure 4:**
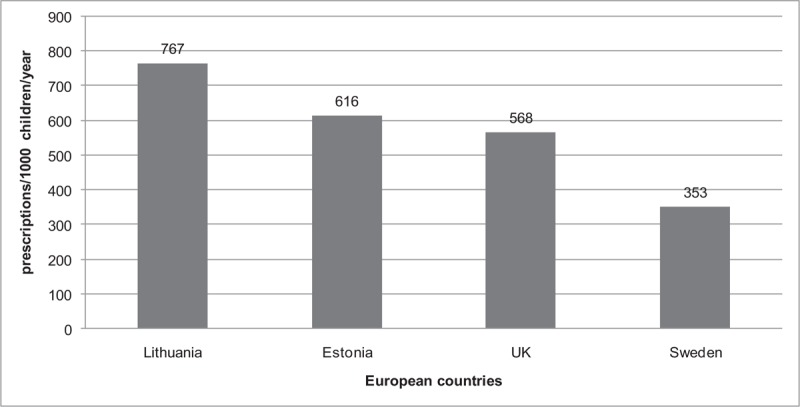
Total antibiotic utilization (prescriptions per 1000 children/year) in 2007 in different European countries.

The European data were identified from the search of published literature reports. Only a few studies with the same methodology were available for comparison with our data. Due to the lack of recent studies, the overall rate of prescribing in Lithuania was compared with the other European countries in 2007. The total antibiotic use in Lithuania was 767 prescriptions /1000 children/year (95% CI 766–778) and it was 19.7% greater than in Estonia, 25.9% higher than in the UK and even 53.9% higher than in Sweden in 2007 (Fig. 4).^[1,11]^

Most of the diseases treated with the antibiotics includes viral infections in our study period. This shows the massive problem of prescribing of antibiotics for children not according to the therapeutic indication but mostly because of fear that the child could develop some kind of serious infection. However, a recent systematic review of the literature stated that not prescribing antibiotics for children with respiratory infections resulted in only an additional 3.8 suppurative complications per 10,000 children.^[14]^ It shows that there is a need for new evidence-based guidelines of prescriptions of antibiotics in Lithuania and another serious problem of not doing enough to diagnose viral infection and reject bacterial.

There were differences observed in the choice of agents between countries because of different spread of bacteria. Penicillins with extended spectrum and combinations of penicillins, including beta-lactamase inhibitors (broad-spectrum penicillins) were prescribed most frequently (number of prescriptions of both subgroups was 387 prescription/1000 children/year) in Lithuania in 2011. We spotted a problem that doctors do not prescribe beta-lactamase sensitive penicillins anymore (29 prescription/1000 children/year) in Lithuania. So, that is why the use of narrow-spectrum penicillins was significantly lower in Lithuania than in Sweden.^[1]^

Excessive use of antibiotics may result in resistance of bacteria to wide spectrum antibiotics and higher possibility of other infections developing and bacterial becoming more and more resistant. In our study macrolides were used excessively in whole period of the study (2003–2012) and mostly were prescribed in 2011 (176 prescription/1000 children/year, 25.25% of all the antibiotics), comparing to Sweden where only 29 prescription/1000 children were prescribed and that matches 8% of all the prescriptions,^[1]^ Estonia (24% of all the prescriptions) and Germany (23.58%).^[1,12]^ High use of macrolides is known to accelerate the rise of macrolide-resistant bacteria.^[12]^ Also, macrolides (for example, clarithromycin) are included in the first-line treatment of *Helicobacter pylori* eradication, thus the growing utilization of macrolides may be related to the increase of clarithromycin-resistant *H pylori* strains and failure of *H pylori* eradication therapy. A study from France showed that in children over 11 years 22.8% of *H pylori* strains were resistant to clarithromycin.^[15]^ Austrian researchers report a constantly rising resistance of *H pylori* to clarithromycin in children, for example, during the last decade it increased up to 34% of all cases.^[15]^ In the study which was done in Lithuania, authors concluded that *H Pylori* is already resistant to macrolides.^[15]^ So this raises a question if the extensive use of macrolides in pediatric age helps *H pylori* become more and more resistant to first line treatment. Our study showed that the utilization of macrolides increased significantly in Lithuania; thus, based on the data from the other countries about the growing resistance of *H pylori* strains, it would be appropriate to control prescriptions of macrolides using national recommendations and/or restrictions in order to avoid the errors in eradication of *H pylori*.

The amount of prescribed broad-spectrum penicillins and macrolides differed significantly in Lithuania compared to Sweden. It might be because Sweden has multilayer national educational and regulatory programs (STRAMA) to control inappropriate antibiotic prescription, meanwhile, there is no similar program in Lithuania.^[1]^ It is important that there is also no similar program in Estonia but somehow, they manage to prescribe less broad-spectrum antibiotics.^[1,12]^

The rate of prescription of cephalosporins was 12.7% of all the prescribed antibiotics during the study period in Lithuania. For instance, cephalosporins were the runner-up for most commonly prescribed drugs in Germany (27%) and the third in Estonia (16%).^[1,12]^ Cephalosporins were hardly ever prescribed for the treatment of children in Denmark and in the Netherlands.^[15,16]^

Overall, our data shows the preference to prescribe extended-spectrum antibiotics more often than narrow-spectrum in the pediatric population in Lithuania. This might be because children are being treated for acute bronchitis (25.6% of prescriptions) and acute pharyngitis (14.6%) with broad-spectrum antibiotics when 90% of the cases is caused by viruses and no antibiotics are needed. There are other studies which have showed similar results – antibiotics are prescribed to treat acute bronchitis, pharyngitis, laryngitis, tracheitis and other viral infections and the use of antibiotics as first-line treatment is not recommended by the guidelines.^[9,10]^

## 5 Conclusion

Most of the diseases that the antibiotics were prescribed includes viral infections in our study period. The most commonly prescribed antibiotics were broad-spectrum. The consumption of antibiotics was evenly increasing during 2003 to 2012 period, but the distribution of separate antibiotic group remained the same.

The least prescribed antibiotics during our period of study was beta-lactamase sensitive penicillins.

Excessive use of antibiotics may result in resistance of bacteria to wide spectrum antibiotics and higher possibility of other infections developing and bacterial becoming more and more resistant.

The study is ongoing, the latter results will be published in 2 years.

## 6 Limitations

Drug utilization data presented DDDs only gives a rough estimate of consumption and not an exact picture of actual use. It enables the researcher to assess trends in trends in drug consumption and to perform comparisons between population groups. Also, we were unable to collect data by groups. Our data provides no information about antibiotic use in hospitals and is insufficient of other illnesses that the child might have developed.

## Author contributions

**Data curation:** Egle Karinauske, Skaiste Kasciuskeviciute.

**Formal analysis:** Vilma Morkuniene.

**Methodology:** Egle Karinauske, Skaiste Kasciuskeviciute, Vilma Morkuniene, Kristina Garuoliene, Edmundas Kadusevicius.

**Resources:** Kristina Garuoliene.

**Supervision:** Edmundas Kadusevicius.

**Writing – original draft:** Egle Karinauske, Skaiste Kasciuskeviciute, Edmundas Kadusevicius.

**Writing – review & editing:** Egle Karinauske, Skaiste Kasciuskeviciute, Kristina Garuoliene, Edmundas Kadusevicius.

Egle Karinauske orcid: 0000-0002-4838-992X.
